# Transient trimethylaminuria related to menstruation

**DOI:** 10.1186/1471-2350-8-2

**Published:** 2007-01-27

**Authors:** Makiko Shimizu, John R Cashman, Hiroshi Yamazaki

**Affiliations:** 1 Laboratory of Drug Metabolism and Pharmacokinetics, Showa Pharmaceutical University, Machida, Tokyo 194-8543, Japan; 2 Human BioMolecular Research Institute, San Diego, CA 92121, USA

## Abstract

**Background:**

Trimethylaminuria, or fish odor syndrome, includes a transient or mild malodor caused by an excessive amount of malodorous trimethylamine as a result of body secretions. Herein, we describe data to support the proposal that menses can be an additional factor causing transient trimethylaminuria in self-reported subjects suffering from malodor and even in healthy women harboring functionally active flavin-containing monooxygenase 3 (FMO3).

**Methods:**

FMO3 metabolic capacity (conversion of trimethylamine to trimethylamine *N*-oxide) was defined as the urinary ratio of trimethylamine *N*-oxide to total trimethylamine.

**Results:**

Self-reported Case (A) that was homozygous for inactive Arg500stop FMO3, showed decreased metabolic capacity of FMO3 (i.e., ~10% the unaffected metabolic capacity) during 120 days of observation. For Case (B) that was homozygous for common [Glu158Lys; Glu308Gly] *FMO3 *polymorphisms, metabolic capacity of FMO3 was almost ~90%, except for a few days surrounding menstruation showing < 40% metabolic capacity. In comparison, three healthy control subjects that harbored heterozygous polymorphisms for [Glu158Lys; Glu308Gly] *FMO3 *or homozygous for wild *FMO3 *showed normal (> 90%) metabolic capacity, however, on days around menstruation the FMO3 metabolic capacity was decreased to ~60–70%.

**Conclusion:**

Together, these results indicate that abnormal FMO3 capacity is caused by menstruation particularly in the presence, in homozygous form, of mild genetic variants such as [Glu158Lys; Glu308Gly] that cause a reduced FMO3 function.

## Background

Trimethylaminuria, or fish odor syndrome, includes a transient or mild malodor caused by an excessive amount of malodorous trimethylamine as a result of body secretions [1,2]. The causal factor of excessive free trimethylamine is reduced enzyme capacity, or maybe substrate overload. The decreased enzyme capacity to form non-odorous trimethylamine *N*-oxide could be a result by an inherited deficiency (primary genetic trimethylaminuria) and/or by hormonal modulation or liver damage (transient trimethylaminuria) [2,3]. For trimethylaminuria, at least 40 genetic polymorphisms of the *flavin-containing monooxygenase 3 *(*FMO3*) gene have been reported [4,5]. For transient trimethylaminuria, a change of metabolic capacity in one individual around the time of menstruation has been reported [6]. Herein, we describe data to support the proposal that menses can be an additional factor causing transient trimethylaminuria in self-reported subjects suffering from malodor and even in healthy women harboring functionally active FMO3.

## Methods

Japanese female volunteers included two subjects suffering self-reported malodor that responded to an Internet article and three healthy laboratory members as controls, ranging from 21 to 37 years of age [5,7]. Written consent was obtained from the individuals for publication of study. FMO3 metabolic capacity (conversion of trimethylamine to trimethylamine *N*-oxide) was defined as the urinary ratio of trimethylamine *N-*oxide to total trimethylamine (% of trimethylamine *N*-oxide/[trimethylamine + trimethylamine *N*-oxide]) determined by GC [7]. The *FMO3 *DNA sequence of genomic DNA prepared from peripheral lymphocytes or buccal cells from the study participants was also analyzed [3,5]. The study participants collected their urine samples using a procedure described previously [7]. The ethics committee of Showa Pharmaceutical University approved this study.

## Results and discussion

**Figure 1:**
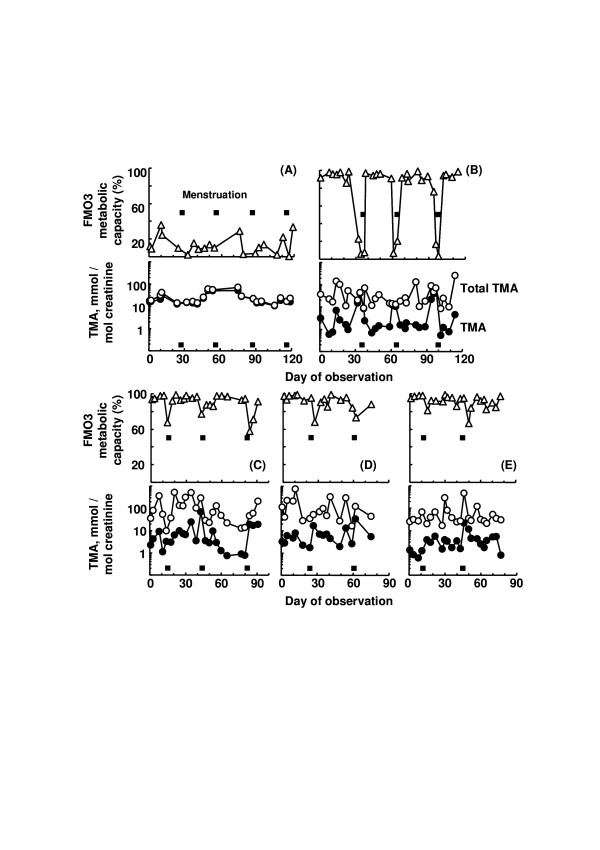
Decreased metabolic capacity of FMO3 (△), as indicated as percent free trimethylamine (TMA, ●) to total trimethylamine (○) excreted in the urine, in relation to menstruation. Filled boxes indicate the period of menstruation. Total urinary trimethylamine and free trimethylamine concentrations were calculated as mmol trimethylamine (TMA)/mol creatinine. Cases (A) and (B) were self-reported subjects suffering from malodour genotyped for homozygotic inactive Arg500stop *FMO3 *and homozygotic common [Glu158Lys; Glu308Gly] *FMO3*, respectively. Cases (C) and (D and E) were control subjects genotyped as heterozygotic for [Glu158Lys; Glu308Gly] *FMO3 *and homozygotic for wild *FMO3*, respectively.

As shown in Figure 1, Case (A) that was homozygous for inactive Arg500stop *FMO3 *[5], showed decreased metabolic capacity of FMO3 (i.e., 13 ± 10 % (mean ± SD, n = 20) of the unaffected metabolic capacity) during 120 days of observation. For Case (B) that was homozygous for common [Glu158Lys; Glu308Gly] *FMO3 *polymorphisms [1-3], metabolic capacity of FMO3 was almost ~90%, except for a few days surrounding menstruation. In comparison, healthy control Case (C) that harbored heterozygous polymorphisms for [Glu158Lys; Glu308Gly] *FMO3 *showed > 90% metabolic capacity and this was greater than the reported unaffected ratio [1,6,7]. However, for Case (C), on days around menstruation the FMO3 metabolic capacity was decreased to ~60–70%. Control Cases (D) and (E) that were homozygous for wild *FMO3 *also showed normal FMO3 metabolic capacity (i.e., ~> 90%), except for days around menstruation. Together, these results indicate that abnormal FMO3 capacity is caused by menstruation particularly in the presence, in homozygous form, of mild genetic variants such as [Glu158Lys; Glu308Gly] that cause a reduced FMO3 function. This would further suggest that sex hormones play a role in the variable regulation of FMO3. Induced FMO3 activity during pregnancy [8] has been reported, in accordance with the present results.

## Conclusion

Menses can be a factor causing transient trimethylaminuria even in healthy women harboring active enzymes. The present information could be useful in relieving the symptoms for transient and/or mild trimethylaminuria for affected females during menstruation.

## Competing interests

The author(s) declare that they have no competing interests.

## Authors' contributions

MS undertook genotyping and some analyses. JRC was involved in the supervision of the project and revised the paper. HY undertook planning and most analyses and drafted the paper. All authors read and approved the final manuscript.

## Pre-publication history

The pre-publication history for this paper can be accessed here:


